# Rheumatoid arthritis, cardiometabolic comorbidities, and related conditions: need to take action

**DOI:** 10.3389/fmed.2024.1421328

**Published:** 2024-07-24

**Authors:** Salvatore Corrao, Luigi Calvo, Annarita Giardina, Ignazio Cangemi, Fabio Falcone, Christiano Argano

**Affiliations:** ^1^Department of Clinical Medicine, Internal Medicine Unit, National Relevance and High Specialization Hospital Trust ARNAS Civico, Di Cristina, Benfratelli, Palermo, Italy; ^2^Department of Health Promotion Sciences, Maternal and Infant Care, Internal Medicine and Medical Specialties [PROMISE], University of Palermo, Palermo, Italy

**Keywords:** rheumatoid arthritis, cardiovascular morbidity, cardiometabolic comorbidities, multimorbidity, multidisciplinary approach, multidimensional management model

## Abstract

Rheumatoid Arthritis (RA) is associated with an increased risk of cardiovascular disease and mortality, however, traditional cardiovascular risk factors do not fully explain this relationship. This high risk of cardiovascular morbidity and mortality in RA has been increasingly acknowledged in past decades, with accumulating evidence that RA is an independent cardiovascular risk factor; RA is also associated with metabolic syndrome, which correlates with disease activity, contributing to the increased prevalence of coronary heart disease in RA patients. Moreover, multimorbidity, including the presence of long-term conditions, impacts adverse clinical outcomes in RA patients, emphasizing the need for holistic management that requires an understanding of shared pathophysiological mechanisms, such as systemic inflammation and immune dysregulation. For all these reasons, the management of RA patients with cardiometabolic comorbidities is a complex endeavor that requires a patient-centered, multidisciplinary approach. In this sense, there is a need to re-evaluate the approach toward a proactive model of care, moving away from a reactive medical paradigm to a multidimensional integrated management model, including aggressive screening, preventive strategies, and tailored therapeutic interventions. The aim of this review was to thoroughly review the literature on cardiometabolic comorbidities and related conditions linked to RA to enable us to identify the necessary actions required to effectively tackle the increasing burden of illness from a fully comprehensive perspective.

## 1 Introduction

The cumulative global burden of autoimmune and inflammatory rheumatic diseases, including of Rheumatoid Arthritis (RA), is substantial, impacting individuals and healthcare systems worldwide and the worldwide age-standardized prevalence rate and years lived with disability have risen over time, with an expected number of cases to keep growing through 2050 (1, 2). For this reason, there is a global necessity for enhanced early detection and management of RA to diminish the impending disease burden. The burden of RA is multifaceted, encompassing the disease’s physical and socioeconomic aspects. RA is associated with an increased risk of cardiovascular disease (CVD), which cannot be fully explained by traditional risk factors, necessitating the development of risk calculators that account for disease activity (3). Inflammation plays a crucial role in atherosclerosis and coronary artery disease, highlighting the intricate relationship between RA and cardiovascular health (4). RA is also associated with metabolic syndrome (MS), which correlates with disease activity, contributing to the increased prevalence of coronary heart disease in RA patients (5). Moreover, multimorbidity, including the presence of long-term conditions, impacts adverse clinical outcomes in RA patients, emphasizing the need for holistic management (6). Given this, frailty is associated with the age-related decline in physiological reserve and is an increasingly important concept in managing chronic diseases, including RA (7). The disease is associated not only with an increased risk of CVD and MS but also with depression, impacting both individual patients and healthcare systems (8). The multifaceted burden of RA underscores the importance of comprehensive management and interventions to address the complex needs of RA patients.

Our aim was to thoroughly review the literature on cardiometabolic comorbidities and related conditions linked to RA. This would enable us to identify the necessary actions required to effectively tackle the increasing burden of illness from a holistic perspective.

Literature review included basic science, cohort studies, intervention and observational trials, and review articles indexed in PubMed.

The following search terms were used: “Rheumatoid Arthritis, cardiovascular morbidity, cardiometabolic comorbidities, multimorbidity, multidisciplinary approach, multidimensional management model.” Inclusion of studies was based on relevance, in accordance with authors’ opinion; studies were gathered in to main topics: (1) The interrelation between RA, cardiometabolic comorbidities and related conditions; (2) Management of Complexity in RA Patients.

## 2 The interrelation between RA, cardiometabolic comorbidities and related conditions

### 2.1 Cardiovascular and metabolic comorbidities

**Figure 1:**
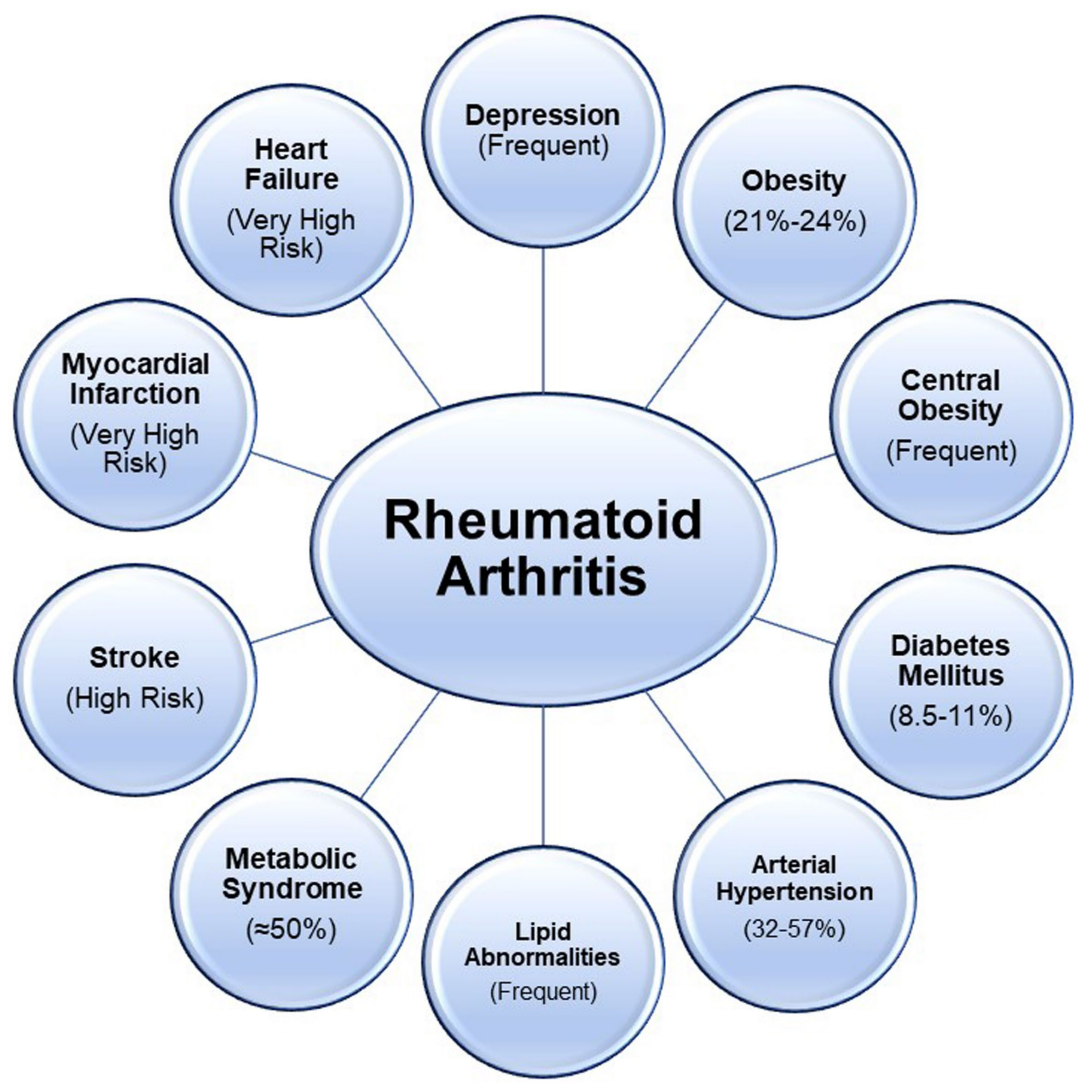
Cardiometabolic and other related conditions associated with Rheumatoid are represented. High risk refers to a hazard ratio greater than 1; very high risk refers to a hazard ratio greater than 2.

The prevalence of cardiovascular comorbidities in RA is a topic of significant interest due to the increased risk of CVD. Several studies have investigated the prevalence of cardiovascular comorbidities in RA, shedding light on the elevated risk of conditions such as hypertension, dyslipidemia, and atherosclerosis in this patient population. Jin et al. (9), using data from The Chinese Registry of Rheumatoid Arthritis, calculated the baseline prevalence rates of major comorbidities, including cardiovascular diseases. The study reported a prevalence rate of cardiovascular diseases in the Chinese AR population at 2.2% (95% CI 2.0–2.5). In another study, Chen et al. (10) discussed the impact of inflammation on cardiovascular risk in RA patients. The study emphasized the association between RA and increased prevalence of cardiovascular comorbidities, such as congestive heart failure, acute myocardial infarction, and atrial fibrillation, indicating the substantial burden of CVD in RA patients. Furthermore, Zegkos et al. (11) discussed in an extensive review the increased cardiovascular morbidity and mortality in RA patients, highlighting the role of cumulative inflammatory burden and antirheumatic medication-related cardiotoxicity as important contributors to the prevalence of cardiovascular comorbidities in RA. Figure 1 shows the level of risk and prevalence of various cardiometabolic conditions and other related ones (8, 12–18).

Another important aspect is represented by the interplay between RA and metabolic conditions. The prevalence of metabolic comorbidities in RA is a topic of significant interest due to the impact of these comorbidities on the overall health and management of RA patients. Several studies have investigated the prevalence of metabolic comorbidities in RA, shedding light on the elevated risk of conditions such as MS, diabetes, and dyslipidemia in this patient population. One study by Dougados et al. (19) reported a high prevalence of metabolic abnormalities, such as hyperglycemia and hyperlipidemia, in RA patients. The study detected abnormalities in vital signs, such as elevated blood pressure, and identified conditions that manifest as laboratory test abnormalities, such as hyperglycemia and hyperlipidemia, indicating the significant burden of metabolic comorbidities in this population. Zhang et al. (20) conducted a meta-analysis of observational studies to assess the prevalence of MS in RA patients. The study reported a high prevalence of MS in RA patients, highlighting the increased risk of metabolic abnormalities in this population. Furthermore, a review by Abdul-Qahar (12) investigated the prevalence of MS in RA patients in Iraq. The study reported a high prevalence of MS in RA patients, indicating the significant burden of metabolic comorbidities in this population. The burden of metabolic comorbidities in RA underscores the importance of comprehensive metabolic risk assessment and management in this patient population. Regarding diabetes mellitus, the prevalence of this condition in RA has been reported in several studies. In particular, it seems to range from 8.5 to 10.4%, exceeding the overall population prevalence of around 3% (21–23). Thus, the prevalence of diabetes in RA patients indicates that diabetes is relatively common in this population. Obesity is another common condition in RA patients with significant metabolic and cardiovascular implications (24), which is a predictive factor of both RA and its activity (25–27). However, it is central obesity, characterized by excess abdominal fat, that is strongly associated with an increased risk of metabolic and cardiovascular diseases. Indeed, Stavropoulos-Kalinoglou et al. (28) discussed the impact of central obesity on cardiovascular risk in RA patients, highlighting the significant metabolic and cardiovascular complications associated with central obesity. Central obesity, high blood pressure, and glycemic and lipid abnormalities (high triglyceride or low HDL-cholesterol blood levels) are all components of the so-called MS. This condition has a high prevalence above 20% in RA patients (29, 30). All these conditions represent a metabolic mix associated with RA and are burdened *per se* by an increased cardiovascular risk.

### 2.2 Cardiovascular outcomes and heart abnormalities

RA is associated with an increased risk of CVD and cardiovascular mortality, accounting for about 30–40% of all deaths in RA (3, 31, 32), leading the European League Against Rheumatism to recommend that CVD risk estimates from conventional calculators be multiplied by 1.5 for RA patients (33). However, traditional cardiovascular risk factors do not fully explain this relationship (34). Several observational studies have identified associations between higher disease activity in RA and cardiovascular outcomes (3). The increased risk of cardiovascular morbidity and mortality in RA has been increasingly acknowledged in past decades, with accumulating evidence that RA is an independent cardiovascular risk factor (35). Patients with RA have an increased risk of developing heart disease, and the pattern of this risk is not entirely clear (36). RA patients have an increased risk of cardiovascular events, and the relative contributions of traditional cardiovascular risk factors and markers of RA severity to CVD are unclear (34, 37). The inflammatory reaction in RA has predictive importance for cardiovascular morbidity (31, 38). Furthermore, RA patients have an increased cardiovascular mortality rate and an increased premature death rate, and they have a higher incidence of atherosclerosis (39); this risk is doubled with age in RA patients (40). Compared with patients without RA, those with RA for at least 10 years have an adjusted relative risk of myocardial infarction of 3.10 (95% CI, 1.64 to 5.87) and those with RA for <10 years had an adjusted relative risk of 1.16 (95% CI, 0.52 to 2.59) (41). The prevalence ratio of atherosclerosis (1.9), peripheral vascular disease (2.4), ischemic heart disease (1.5) heart failure (2.0), cerebrovascular disease (1.6), DMT2 (1.4), lipid abnormalities (1.2), and arterial hypertension (1.3) are higher in RA patients than healthy controls (42). Interestingly, V P van Halm et al. demonstrated that the risk of CVD associated with RA (2.7–95% CI 1.2 to 5.9) equals DMT2 patients’ risk, after adjustment for cardiovascular risk factors cardiovascular risk factors (43). Even though the paucity of data on the outcomes Acute Coronary Syndromes (ACS) in patients with RA in the contemporary era, a study based on the Nationwide Inpatient Sample (NIS) database from 2002 to 2016 analyzed 9,482,509 ACS from 2002 to 2016. That study showed that there was a rise in the number of hospitalizations with acute myocardial infarction (mainly driven by NSTEMI) in RA patients (6,730 in 2002 vs. 10,829 in 2016, Ptrend<0.001) compared with a downtrend in RA patients (Ptrend = 0.01). On the other hand, the overall in-hospital mortality was 5.4% (downtrend: 5.8% in 2002 versus 5.2% in 2016, Ptrend = 0.01) (44). In summary, the relationship between RA and cardiovascular outcomes is well-established. The inflammatory reaction in RA has predictive importance for cardiovascular morbidity, therefore, it is crucial to consider and manage cardiovascular risk factors in patients with RA. In the past, our research group has significantly contributed to the development of knowledge in understanding cardiovascular alterations in RA patients. In a study published in the European Journal of Clinical Investigation (45), we investigated the presence of left ventricular filling abnormalities in patients with RA who did not have clinically evident CVD. The study found that the excess of cardiovascular mortality might be related to heart failure and not to coronary heart disease or hypertension. This research shed light on the specific cardiovascular complications experienced by RA patients, emphasizing the importance of understanding and addressing these issues to improve patient outcomes. Our work has contributed to the growing body of evidence highlighting the intricate relationship between RA and cardiovascular health (46). In particular, AR patients have left ventricle diastolic dysfunction, valvular alterations, aortic Valsalva aneurysms and left ventricular hypertrophy not related to blood pressure (45, 47–50). All these specific cardiac abnormalities shape the picture of rheumatoid myocardiopathy.

On the other hand, drugs play an important role in cardiovascular mortality. Rho et al. (51) discusses the concern that corticosteroids, non-steroidal anti-inflammatory drugs, and COX-2 inhibitors could affect cardiovascular risk adversely in patients with RA. He also mentions that drugs such as the antimalarial hydroxychloroquine may have beneficial effects.

### 2.3 Periodontal disease

The relationship between RA and periodontal disease has been a subject of interest in various studies. Several studies shed light on this relationship. Mercado et al. (52) investigated the relationship between RA and periodontitis, providing evidence of a significant association between the two conditions. In particular, this study found that swollen joints, health assessment questionnaire scores, levels of C-reactive protein, and erythrocyte sedimentation rate were the principal variables associated with periodontal bone loss.

Similarly, González-Febles and Sanz (53) investigated the effect of different RA medications on periodontal status, revealing that these treatments arrested periodontal inflammation irrespective of periodontitis severity. Furthermore, a systematic review with metanalysis by Kaur et al. (54) provided evidence based on biochemical markers and clinical variables, suggesting a potential association between RA and periodontal disease. Moreover, Bartold et al. (55) discussed the relationship between periodontitis and RA, highlighting that individuals with advanced RA are more likely to experience more significant periodontal problems compared to their non-RA controls.

Additionally, the systematic review with meta-analysis by Buwendo et al. (56) pointed out a significant relationship between periodontal disease and RA, with increased periodontal pocket depth and clinical attachment loss. Furthermore, the study by Zhao et al. (57) suggested that the presence of periodontal disease might contribute to the progression of RA. At the same time, RA might have little effect on accelerating the development of periodontal disease.

### 2.4 Thromboembolic venous disease

RA has been associated with an increased risk of thromboembolic venous disease, including deep vein thrombosis (DVT) and pulmonary embolism (PE). Several studies have investigated the relationship between RA and thromboembolic events, highlighting the elevated risk of venous thromboembolism (VTE) in patients with RA compared to the general population (58–60). The risk of VTE in RA patients has been found to be significantly increased, with a higher incidence of DVT and PE (58, 60). Additionally, the presence of elevated rheumatoid factor has been associated with an increased long-term risk of developing RA and an increased risk of VTE (61). Furthermore, the use of disease-modifying antirheumatic drugs (DMARDs), both biological and non-biological in comparison with methotrexate, in RA patients has been studied in relation to the risk of VTE, with research suggesting that initiating DMARDs may impact the risk of VTE in RA patients (62). However, some studies have reported conflicting findings, with certain investigations indicating that anti-TNF therapy does not increase the risk of VTE in RA patients (63).

A recent study examined the likelihood of VTE, PE, and DVT in patients newly diagnosed with RA compared to the general population, monitoring these risks for up to 5 years after diagnosis (64). The research utilized a comprehensive administrative health database from British Columbia, Canada, encompassing 39,142 patients with a new RA diagnosis. Findings revealed that these patients experienced higher rates of VTE, PE, and DVT than those without RA, excluding thromboembolism types. This was true even when adjusting for known VTE risk factors, with RA patients showing elevated hazard ratios. In contrast, Gazitt et al. (65) found no heightened VTE risk in patients with Psoriatic Arthritis after conducting a multivariable analysis in a separate population study. This suggests that the risk of VTE may be specific to AR, and Janus kinase inhibitors may influence this increased VTE risk among RA patients. Corrao S., one of the authors of this review, recently published a point of view on this important issue (46). The “lipid paradox” is a phenomenon in active RA and other inflammatory diseases where low LDL levels correlate with higher cardiovascular risk. Literature suggests this paradox stems from inflammation-induced alterations in cholesterol metabolism. Proinflammatory cytokines promote the uptake of LDL by the liver, decreasing circulating LDL. This paradox suggests that lipid quality, particularly HDL function, in RA may contribute more to CVD risk than lipid quantity. In the context of RA treatment, reducing inflammation paradoxically increases both LDL and HDL levels, yet this does not seem to correlate with more cardiovascular events. A study by Fernández-Ortiz AM et al. (66), on early arthritis, indicated that high disease activity was linked to lower total cholesterol and LDL but higher oxidized LDL, which may explain the increased cardiovascular risk in RA with lower LDL levels. While there is no clear evidence linking the lipid paradox to VTE events, dyslipidemia’s association with VTE is established. Studies have linked higher LDL and, in women, triglycerides to VTE risk (67). Moreover, statin use has been correlated with a reduced risk of recurrent PE and lower cardiovascular events and mortality (68). RA itself is a risk factor for VTE, which may even manifest before RA onset (69). Nationwide studies have confirmed higher DVT and PE risks in RA patients than in the general population (70). Data from phase II and III trials of upadacitinib in RA and Psoriatic Arthritis patients show insufficient management of key cardiovascular and VTE risk factors in rheumatological care (71). These findings highlight the necessity of managing increases in LDL cholesterol, considering the U-shaped relationship with cardiovascular risk. Clinicians must recognize the role of LDL in atherosclerotic CVD and ensure rigorous control of LDL levels in all patients.

## 3 Management of complexity in RA patients

The interrelation between RA, cardiometabolic comorbidities and related conditions (Figure 1) complicates clinical management and requires understanding shared pathophysiological mechanisms, such as systemic inflammation and immune dysregulation. Although there have been substantial improvements in the control of inflammation in RA patients over 20 years, poor control of traditional CV risk factors might be the main reason for the increased risk of CVD (72). These comorbidities contribute to the heightened cardiovascular risk in RA patients, necessitating aggressive screening, preventative strategies, and tailored therapeutic interventions. We previously published the approach to clinical complexity in another chronic condition, diabetes mellitus (73). The challenges and strategies in managing the complexity of RA patients with concurrent cardiometabolic disorders and related conditions are fundamentally the same beyond the rheumatological specificity. To facilitate the seamless integration of healthcare services and foster efficient communication between community care providers and tertiary care centers, the adoption of clinical complexity levels should be prioritized over the traditional focus on care intensity levels (73–75). Then, when devising care plans, it is essential to categorize patients according to the progression stages of their disease, linked with the requisite complexity of care according to the Kaiser Permanente Pyramid that stratifies patients into specific subgroups grounded on the developmental phase of their respective illnesses (76). Moreover, starting from these foundation principles, there is a need to re-evaluate the approach toward a proactive model of care, moving away from a reactive medical paradigm to a multidimensional integrated management model.

Key components of an integrated care management model include:

- Establishment of an interdisciplinary team focused on RA and its related complications, adopting a holistic approach to equip healthcare professionals with the skills necessary for chronic disease management.
- Implementation of a diagnostic and therapeutic protocol that encompasses collaboration between general practitioners, rheumatologists, diabetologists, and other medical specialists, like physiatrists and surgeons, nurses, dietitians, psychologists, social workers, health administrators, pharmacists, and representatives from patient organizations.
- Proactive engagement of individuals with RA in their treatment journey, through the selection of patients who are candidates for integrated management and the organization of educational, preventative, and therapeutic initiatives. This ensures tailored support from various healthcare providers.
- Development of an information system to identify the target population and evaluate processes and outcomes. This system should have the capability to actively recall patients into the care continuum and assist healthcare providers in the efficient and timely exchange of critical patient management information.
- Coordination among all the stakeholders involved in the care process, including physicians, healthcare workers, pharmacists, and professionals in residential and home care settings, all within the framework of the local health district.

### 3.1 Screening and risk assessment

- Regular screening for cardiometabolic risk factors is imperative in RA management. This includes monitoring blood pressure, lipid profiles, fasting glucose levels, and body mass index. Risk assessment tools should be adapted to account for the inherent risk associated with RA. In fact, traditional risk factors alone do not fully account for the excess cardiovascular risk and mortality in RA patients (32, 36, 77). Early identification of comorbid conditions is essential to mitigate the risk of cardiovascular events, and stringent control of LDL-cholesterol levels is needed (3).
- CVD risk prediction algorithms should be adapted considering a 1.5 multiplication factor, if this is not already included in the risk score (33). Early identification of a severe chronic inflammatory process (presence of autoantibodies, high disease activity, early radiographic joint damage, treatments non-responder) might be related with higher cardiovascular risk and may need a closer follow-up; however, evidences are not yet strength in this field. Internists (Physicians) should play a key role in the interdisciplinary team coordination and in involvement of other specialists in order to face the specific pattern of manifestations of the disease of each patient and to help the rheumatologist in therapy reconciliation.

### 3.2 Pharmacological management

The pharmacological treatment of RA patients with cardiometabolic comorbidities involves a delicate balance. Anti-inflammatory agents, particularly DMARDs and biologics, can exert differential impacts on cardiometabolic profiles. For instance, methotrexate and TNF inhibitors may have cardioprotective effects, whereas glucocorticoids can exacerbate cardiometabolic dysregulation. Therefore, the choice of RA therapy must consider the patient’s cardiometabolic status. We believe that internists should be engaged in the Therapeutical reconciliation of metabolic and/or cardiovascular and other therapies.

### 3.3 Lifestyle modifications

Lifestyle interventions are a cornerstone in managing cardiometabolic comorbidities in RA patients. These include smoking cessation, adopting a heart-healthy diet, engaging in regular physical activity, and achieving weight control. Such interventions can ameliorate both RA symptoms and cardiometabolic parameters. In particular, our findings (78) showed that combining physical exercise and occupational therapy positively affects patients’ quality of life with RA, considering disease activity, global health status, and mental health.

## 4 Conclusion

**Figure 2:**
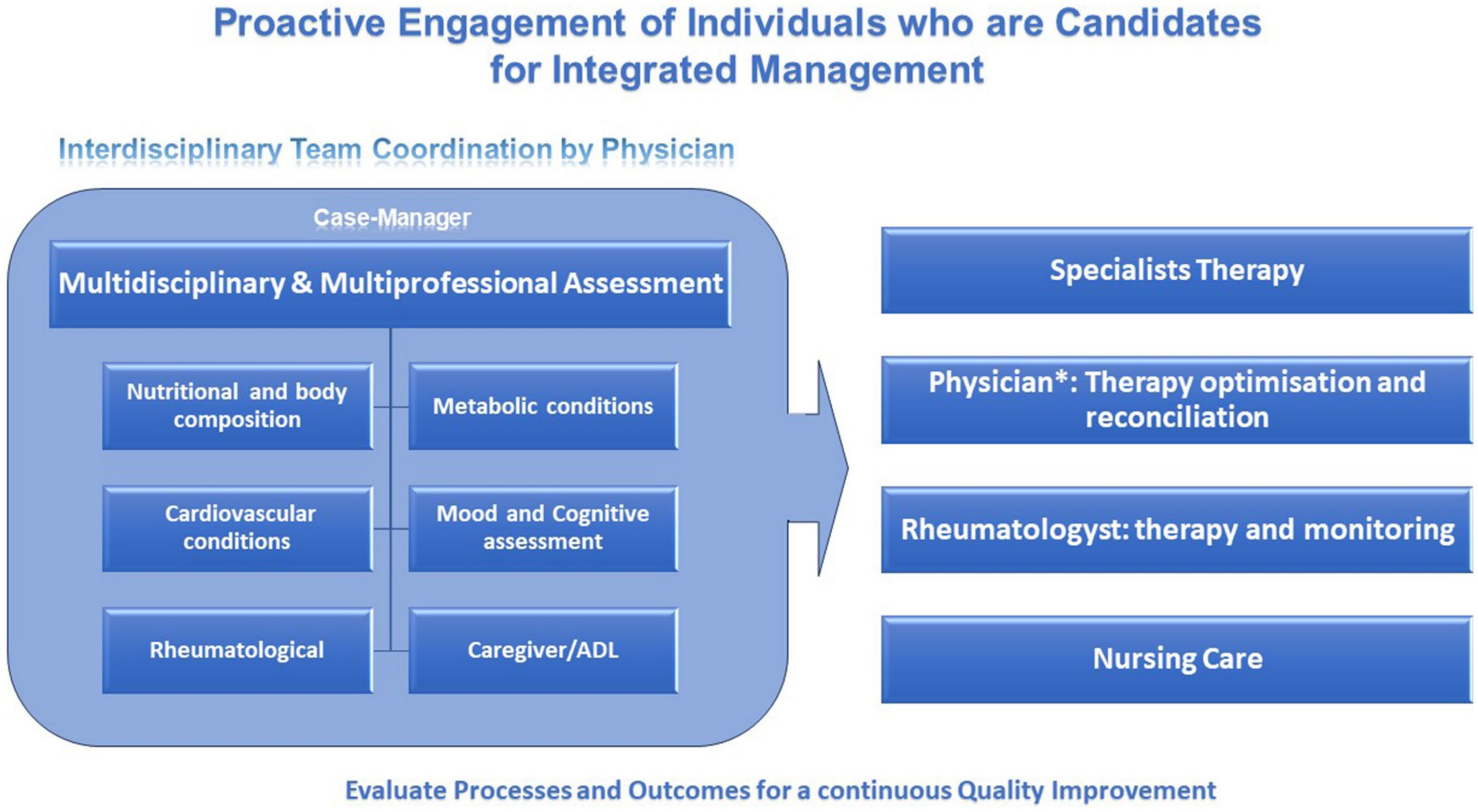
Summary diagram of patient-centered, multidisciplinary approach of the health-care plan of RA patients. *Internist (Physician), plays a key role in the interdisciplinary team coordination and in involvement of other specialists in order to face the specific pattern of manifestations of the disease of each patient and to help the rheumatologist in therapy reconciliation.

The management of RA patients with cardiometabolic comorbidities is a complex endeavor that requires a patient-centered, multidisciplinary approach (Figure 2). Optimized management includes vigilant screening, careful selection of pharmacotherapy, and robust lifestyle modification strategies. The awareness and the right approach to complexity, using a proactive model of medicine through a multidimensional integrated management process flow, appears to be a win-to-win model.

## Author contributions

SC: Conceptualization, Data curation, Formal analysis, Methodology, Project administration, Supervision, Validation, Visualization, Writing – original draft, Writing – review & editing. LC: Conceptualization, Writing – review & editing. AG: Conceptualization, Writing – review & editing. IC: Conceptualization, Writing – review & editing. FF: Conceptualization, Writing – review & editing. CA: Conceptualization, Writing – review & editing.
